# Oxygen-Linked S-Nitrosation in Fish Myoglobins: A Cysteine-Specific Tertiary Allosteric Effect

**DOI:** 10.1371/journal.pone.0097012

**Published:** 2014-05-30

**Authors:** Signe Helbo, Andrew J. Gow, Amna Jamil, Barry D. Howes, Giulietta Smulevich, Angela Fago

**Affiliations:** 1 Department of Bioscience, Aarhus University, Aarhus, Denmark; 2 Department of Pharmacology and Toxicology, Ernest Mario School of Pharmacy, Rutgers University, Piscataway, New Jersey, United States of America; 3 Department of Chemistry “Ugo Schiff”, University of Firenze, Sesto Fiorentino (FI), Italy; National Research Council of Italy (CNR), Italy

## Abstract

The discovery that cysteine (Cys) S-nitrosation of trout myoglobin (Mb) increases heme O_2_ affinity has revealed a novel allosteric effect that may promote hypoxia-induced nitric oxide (NO) delivery in the trout heart and improve myocardial efficiency. To better understand this allosteric effect, we investigated the functional effects and structural origin of S-nitrosation in selected fish Mbs differing by content and position of reactive cysteine (Cys) residues. The Mbs from the Atlantic salmon and the yellowfin tuna, containing two and one reactive Cys, respectively, were S-nitrosated *in vitro* by reaction with Cys-NO to generate Mb-SNO to a similar yield (∼0.50 SH/heme), suggesting reaction at a specific Cys residue. As found for trout, salmon Mb showed a low O_2_ affinity (*P*_50_ = 2.7 torr) that was increased by S-nitrosation (*P*_50_ = 1.7 torr), whereas in tuna Mb, O_2_ affinity (*P*_50_ = 0.9 torr) was independent of S-nitrosation. O_2_ dissociation rates (*k*_off_) of trout and salmon Mbs were not altered when Cys were in the SNO or *N*-ethylmaleimide (NEM) forms, suggesting that S-nitrosation should affect O_2_ affinity by raising the O_2_ association rate (*k*_on_). Taken together, these results indicate that O_2_-linked S-nitrosation may occur specifically at Cys107, present in salmon and trout Mb but not in tuna Mb, and that it may relieve protein constraints that limit O_2_ entry to the heme pocket of the unmodified Mb by a yet unknown mechanism. UV-Vis and resonance Raman spectra of the NEM-derivative of trout Mb (functionally equivalent to Mb-SNO and not photolabile) were identical to those of the unmodified Mb, indicating that S-nitrosation does not affect the extent or nature of heme-ligand stabilization of the fully ligated protein. The importance of S-nitrosation of Mb *in vivo* is confirmed by the observation that Mb-SNO is present in trout hearts and that its level can be significantly reduced by anoxic conditions.

## Introduction

S-nitrosation is a widespread and reversible post-translational protein modification in which formally a nitric oxide (NO) molecule is covalently bound to a reactive thiol on a specific cysteine (Cys) yielding S-nitrosothiol (SNO). In analogy with other post-translational modifications such as phosphorylation, SNO formation often induces changes in protein function (reviewed in [1]). Human hemoglobin (Hb), the O_2_ carrier of the blood, was one of the first proteins discovered to be specifically S-nitrosated at Cysβ93 to generate Hb-SNO [2], [3]. This protein modification increases the heme oxygen (O_2_) affinity (*P*_50_, the O_2_ tension at half-saturation) [4], [5] by shifting the allosteric equilibrium between two alternative tetrameric quaternary structures of the Hb. Conversely, the shift in the opposite direction by a decrease in O_2_ levels destabilizes the S-NO bond causing the release of the NO moiety, which may induce vasodilation and increase local blood flow in hypoxic tissues [2], [3], [6].

In being a monomeric protein, myoglobin (Mb), that facilitates O_2_ diffusion from the blood into skeletal and heart muscle of vertebrates [7], [8], has long been believed to function without allosteric control. We recently found that rainbow trout Mb-SNO (i.e. Mb with a reactive Cys in the S-nitrosated form) has a higher O_2_ affinity than the unmodified protein [9]. This effect is unprecedented among Mbs and may be described as a tertiary allosteric effect that is dependent on a covalent SNO modification as opposed to the quaternary allosteric mechanisms of Hb, which involves primarily non covalent interactions with anionic cofactors. Regardless of the molecular mechanism, the direction of change of O_2_ affinity caused by S-nitrosation is the same in trout Mb and in human Hb [2], [9]. We propose that O_2_-linked NO release from Mb-SNO may function to down-regulate respiration in the hypoxic myocardium of trout during intense activity of this fish species [9], [10]. Thus, interactions between NO and the protein moiety of Mb may improve NO-dependent myocardial efficiency and fine-tune the balance between O_2_ demand and aerobic availability in the fish heart [11]. In addition, the extent of NO scavenging by deoxy heme appears to be limited in the hypoxic myocardium of trout, suggesting a role of endogenously produced NO in the regulation of myocardial O_2_ consumption [12].

**Figure 1:**
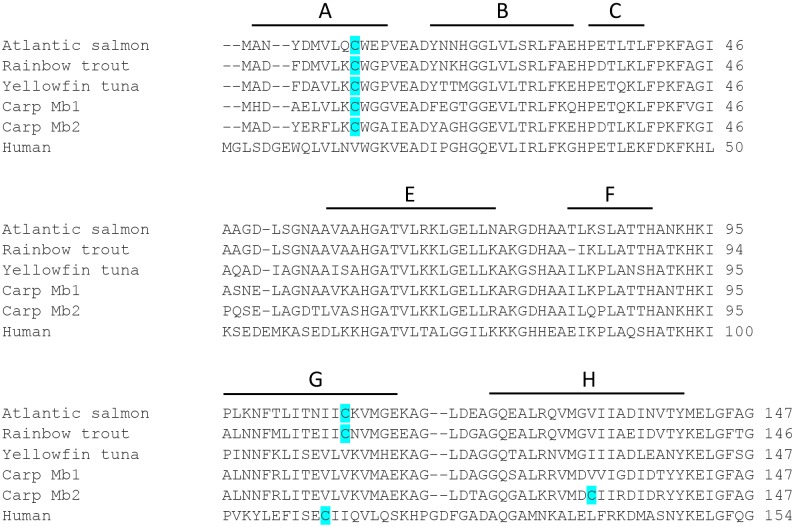
Amino acid sequence alignment of salmon, trout, tuna, carp Mb1 and Mb2 and human Mb shows variable number and position of Cys residues (highlighted). Mb sequences have been retrieved from Pub Med (Atlantic salmon: Atlantic salmon: GenBank, ACM09229.1, rainbow trout: GenBank, BAI45225.1, yellowfin tuna: GenBank: AAG02112.1, common carp Mb1: UniProtKB/Swiss-Prot, P02204.2, common carp Mb2: GenBank: ABC69306.1, human: NCBI Reference Sequence: NP_976311.1). The positions of the α-helices (A–C, E–H) are indicated and are based on the structure of yellowfin tuna Mb [18].

Other studies have reported that human Mb, which contains one Cys residue (Cys110) (Figure 1), can be S-nitrosated and the bound NO can be released by addition of Cu^2+^ and induce vasodilation *in vitro* [13], although it is unknown whether the NO release is dependent on heme oxygenation state, as in trout Mb. However, Cys residues are only found in a few mammalian Mbs and therefore a general physiological role for this mechanism in mammals is unlikely. In contrast, most fish and reptilian Mbs contain one or two Cys at conserved positions suggesting a potential greater functional significance in these animal groups [9], [14]–[17]. Using crystallographic analyses, Schreiter and coworkers [18] have shown that S-nitrosation of Cys10 in blackfin tuna Mb causes structural changes in the protein that could potentially alter heme reactivity. However, functional properties of tuna Mb-SNO have not been measured. In the two Mb isoforms from the common carp (Mb1 and Mb2), we found no effect of S-nitrosation on O_2_ binding [14], and therefore it remains unclear whether the SNO-dependent allosteric regulation of Mb is limited to trout or whether it is more widespread among fish.

In this study, we examine whether Mbs from two other fish species, the yellowfin tuna and the Atlantic salmon, containing one (Cys10) and two Cys residues (Cys10 and Cys107), respectively (Figure 1), undergo changes in O_2_ affinity upon S-nitrosation similar to those described in rainbow trout Mb, containing Cys10 and Cys107 (Figure 1). To this end, we purified Mb from yellowfin tuna and Atlantic salmon and measured reactive thiols, extent of *in vitro* S-nitrosation and O_2_ equilibrium curves and O_2_ dissociation kinetics before and after S-nitrosation. Furthermore, we used UV-Vis and resonance Raman (RR) spectroscopy to investigate possible structural changes of the heme cavity of trout Mb after reacting Cys with *N*-ethylmaleimide (NEM), that yields a more stable and non-photolabile complex than Mb-SNO, and examined the functional properties of trout Mb-NEM in comparison with those of Mb-SNO. Finally, using the biotin switch assay, we have examined levels of Mb-SNO in the trout heart.

## Materials and Methods

Atlantic salmon (*Salmo salar*) were caught by anglers at the stream Lilleåen, Denmark (GPS coordinates: X 542.950,6796 m; Y 6.248.248,3107 m), with a permit from the local sport fishing association Bjerringbro Sportsfiskerforening. Rainbow trout (*Oncorhynchus mykiss*) were obtained from Funderholme trout farm, Denmark. Neither species is endangered or protected. This study was carried out in strict accordance with the current legislation for animal experiments in Denmark (Dyreforsøgsloven nr. 1306, November 23^rd^ 2007, amendments § 1 nr. 612, June 14^th^ 2011). As the research did not included living or anesthetized animals, no ethical approval was necessary. To minimize suffering, fish were killed by a quick blow to the head followed by severing of the spinal cord. Hearts were dissected out and frozen until further use. Frozen yellowfin tuna (*Thunnus albacares*) steaks were obtained from the local fish market. Purified Mb from human heart was bought from Sigma-Aldrich (product number: M6036). All water used was milli-Q grade. Amino acid sequences of Mbs were obtained from the GenBank database (yellowfin tuna: ACM09229.1; Atlantic salmon: AAG02112.1; rainbow trout: BAI45225.1; human: AAH14547.1).

### Purification of Mbs

Mb from salmon and trout hearts was purified as described in detail previously for rainbow trout Mb [9]. Yellowfin tuna Mb was purified from homogenized skeletal muscle. Samples were centrifuged for 25 min at 12,000 g, passed on a PD-10 column (GE Healthcare) equilibrated with 50 mM Tris-HCl, 0.5 mM EDTA, 3 mM dithiothreitol (DTT), pH 8.3 and loaded on a gel-filtration Tricorn Superdex 75 10/300 GL FPLC-column (GE-Healthcare) equilibrated with 50 mM Tris-HCl, 0.5 mM EDTA, 3 mM DTT, 0.15 M NaCl, pH 8.3. Finally, the sample was dialysed against 20 mM Tris-HCl pH 9.2, passed through an ion exchange Hitrap Q-FF FPLC-column, and eluted with a 30-min gradient of 0–0.5 M NaCl in 20 mM Tris-HCl pH 9.2 at a flow rate of 1 mL/min. The purity of salmon and tuna Mb (>95%) was assessed spectrophotometrically from the Soret (416 nm) to protein (280 nm) absorbance peak ratio (A_416_/A_280_>5) and by isoelectrofocusing and SDS electrophoresis on polyacrylamide gels [9].

### Thiol Reactivity

Reactive thiols (-SH/heme) of the Mbs were measured by following the thiol-mediated conversion of 4,4′-dithiodipyridine (4-PDS) into 4-thiopyridone (4-TP, ε_324_ = 19.8 mM^−1^cm^−1^) at 324 nm [19] over time in 50 mM Hepes, pH 7.2, 20°C, at a protein concentration of ∼10 µM, as previously described [9]. A molar ratio of 4∶1 4-PDS/heme was used. Reaction rates were obtained by mono-exponential (tuna and human Mb) or double exponential (salmon Mb) fitting to the absorbance traces.

### Blocking Reactive Thiols

To measure the effect of Cys modification on the O_2_ equilibria and kinetics, trout Mb was reacted with *N*-ethylmaleimide (NEM) at a 3∶1 NEM/heme molar ratio for 1 hour at room temperature (in 50 mM Hepes, pH 7.2) to generate Mb-NEM, which is a non-photolabile and more stable derivative than Mb-SNO. Excess NEM was removed using a PD-10 desalting column (GE-Healthcare). To verify that thiols had reacted with NEM, the amount of free thiols in Mb-NEM was measured with the 4-PDS assay described above.

### Synthesis and Quantification of S-nitrosated Mb

S-nitrosation of Mb to generate Mb-SNO was performed by transnitrosation reaction as previously described for trout Mb [9], in which the NO group is transferred from a S-nitrosated thiol present in excess (i.e. Cys-NO) to free thiols on the Mb protein. In brief, tuna and salmon oxy Mb (in 50 mM Tris-HCl, 0.5 mM EDTA, 0.15 M NaCl, pH 8.3) were incubated with a 2-molar excess of Cys-NO (50 mM in 0.25 M HCl, 0.1 mM EDTA) for 15 min in darkness, at room temperature. Excess Cys-NO was then quickly removed by passage through a PD-10 desalting column (GE Healthcare) equilibrated with 50 mM Tris-HCl, 0.5 mM EDTA, pH 8.3. The SNO/heme yield was measured by the Saville assay [20], as described previously [9].

### O_2_ Binding Equilibria

O_2_ equilibria of Mbs (native, SNO and NEM forms) were measured spectrophotometrically (∼150 µM Mb in 50 mM Tris-HCl, 0.5 mM EDTA pH 8.3 at 20°C, n = 3) in 4 µL samples using a modified thin-layer diffusion chamber technique described in detail elsewhere [9], [21]. O_2_ affinity (*P*_50_, the *P*O_2_ at 50% saturation) and cooperativity were calculated from the zero intercept and slope, respectively of Hill plots: log*Y*/(1-*Y*) vs. log*P*O_2_, where *Y* is the fractional O_2_ saturation. Experiments typically included 4–6 saturation steps.

To verify that the change in O_2_ affinity caused by S-nitrosation was reversible when SNO was removed, CuSO_4_ (1 mM) was added to the salmon Mb-SNO sample to release NO from SNO. However, addition of CuSO_4_ readily oxidized Mb, likely caused by the released NO. Oxidized samples were therefore subsequently incubated with DTT (∼6 mM) on ice overnight to reduce heme and Cys before measuring O_2_ equilibria.

### O_2_ Kinetics

O_2_ dissociation rates of Mbs (native, SNO and NEM forms) were measured at 20°C using an OLIS RSM 1000 UV/Vis rapid-scanning stopped flow spectrophotometer (OLIS, Bogart, CA) coupled to a computer with OLIS data collection software, as previously described [22]. In brief, oxy Mb (∼10 µM heme in 100 mM Tris, 0.5 mM EDTA pH 8.3) was mixed 1∶1 with deoxygenated buffer containing 40 mM dithionite and the change in absorbance after mixing was recorded until reaction completion (<0.1 s) at three wavelengths (418, 420 and 430 nm, n = 5–8). O_2_ dissociation rate constants (*k*_off_, s^−1^) were obtained from averaging monoexponential fittings to the absorption traces at the three wavelengths. O_2_ dissociation equilibrium constants in solution (*K_d_*, µM^−1^) were calculated by multiplying *P*_50_ values (torr) with the solubility coefficient of O_2_ at 20°C in a 100 mM buffer (corresponding to a salinity of 5.84 ‰), which equals 1.77 µM^−1^ torr^−1^ [23]. Apparent O_2_ association rates (*k*_on_, µM^−1 ^s^−1^) were estimated from measured values of *K_d_* and *k*_off_ using the relationship *k*_on_ = *k*_off_/*K*.

### UV-Vis and Resonance Raman Spectroscopy of Trout Mb-NEM

The NEM complex of trout Mb was prepared by addition of 0.2 M NEM stock solution to metMb (390 µM in 0.05 M Hepes, pH 7.2) to achieve a 3∶1 NEM:Mb molar ratio. The solution was carefully mixed and left to incubate at 4°C for 1 h before passing down a Sephadex G25 (Bio Gel P6-DG) desalting column to remove excess NEM. Purified Mb-NEM was immediately placed at −20°C to be used when required. All samples (deoxy, CO and oxy) were prepared from the met form using freshly defrosted Mb-NEM as described elsewhere [24]. The resonance Raman samples were cooled by a gentle flow of N_2_ gas (ca. 12°C) and each individual sample was studied for less than 2 h (usually less than 60–90 min depending on the particular form) to avoid possible degradation of the Mb-NEM complex. If necessary, to improve spectral quality, a new sample was prepared. The UV-Vis and resonance Raman spectra were recorded as previously reported [24].

### Biotin Switch Assay

Prior to measuring Mb-SNO in trout heart with the biotin switch assay, electrically-stimulated ventricle myocardial rings were exposed to either 50% O_2_ or 0% O_2_ (with N_2_ as balance gas) using an experimental protocol described in detail earlier [9], [25]. Experiments were performed in Hepes-Tris buffered trout Ringer solution containing (mM): 115 NaCl, 2.5 KCl, 1 MgSO_4_, 1.5 CaCl_2_, 5 Hepes-Tris, pH 7.5 at 15°C. Ring preparations from a single ventricle heart were allowed to stabilize for 30 min at 50% O_2_, before being exposed for 45 min to either 100% N_2_ or to 50% O_2_ in parallel setups. Ventricle preparations were then quickly taken out, frozen in liquid N_2_ and stored at −80°C. Samples in all three conditions, i.e. 1) 30 min at 50% O_2_ (control), 2) 30 min at 50% O_2_ and 45 min at 100% N_2_, and 3) 30+45 min at 50% O_2_, were defrosted simultaneously and lysates prepared and analysed by the biotin switch method as previously described [26]. 5 ng of total myocardial protein was utilized for the assay. In parallel total protein load was confirmed by silver stain. Total Mb content from the silver stain and Mb-SNO were quantified using densitometry and the Image J software program. The quantity of Mb-SNO was normalized to the total Mb loaded and compared between groups.

### Statistics

Significant differences were evaluated by unpaired t-tests. Statistical significance was accepted at the 95% confidence interval (*P*<0.05). Numeric values are presented as means ± SD unless otherwise stated.

## Results

**Figure 2:**
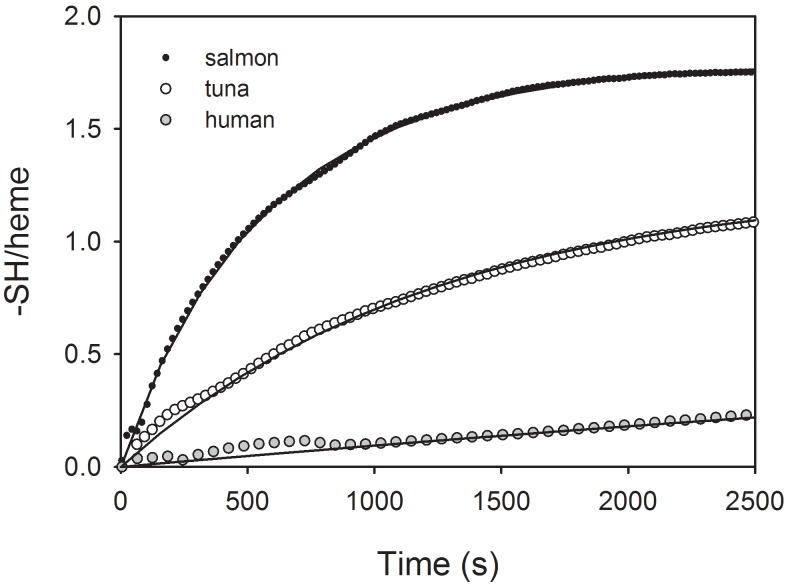
Salmon and tuna Mbs have faster reacting Cys than human Mb. Time course of the reaction of 4-PDS with accessible free thiols of salmon (black dots), tuna (white circles) and human Mb (grey circles) were measured in 50 mM Hepes, pH 7.2 at 20°C at a ratio of 4∶1 4-PDS/heme. Data fittings by double (salmon) or single (tuna and human) exponential equations are indicated.

The 4-PDS assay showed that tuna Mb had ∼1.2, salmon Mb had ∼1.8 and human Mb had ∼1 accessible thiol per heme (Figure 2). This finding is consistent with their amino acid sequences, which show that yellowfin tuna Mb has one Cys (Cys10), Atlantic salmon Mb has two Cys (Cys10 and Cys108) and human Mb has one Cys (Cys110) (Figure 1). Reactions with 4-PDS were faster for salmon Mb (6×10^−3^ and 2×10^−3 ^s^−1^) and tuna Mb (8.1×10^−4 ^s^−1^) than for human Mb (9.7×10^−5 ^s^−1^) (Figure 2), indicating higher thiol reactivity in these fish Mbs than in human Mb. In previous experiments we found that rainbow trout Mb has ∼2 accessible Cys [9] that react with 4-PDS at a rate (5×10^−3 ^s^−1^) similar to that found here for salmon Mb.

Salmon and tuna Mb could be S-nitrosated *in vitro* by transnitrosation reaction with a 2∶1 ratio of Cys-NO to heme, both with a yield of ∼0.5 SNO/heme, as measured by the Saville method. However, we were unable to obtain S-nitrosated human Mb using the protocol described by Rayner and coworkers [13], at least not to an extent detectable by the Saville method, probably because of the very low thiol reactivity of this Mb (Figure 2). Therefore, no further experiments could be performed on human Mb. Some inevitable partial heme oxidation of salmon and tuna Mb-SNO occurred, which, however, did not impair oxygenation of the remaining ferrous Mb, as also described earlier for trout Mb-SNO [9]. Attempts to increase the yield of S-nitrosation in salmon and tuna Mbs by increasing incubation times with Cys-NO or Cys-NO to heme ratios were unsuccessful because of considerable heme oxidation, generated from the reaction between oxygenated heme and the NO released over time by Cys-NO.

**Figure 3:**
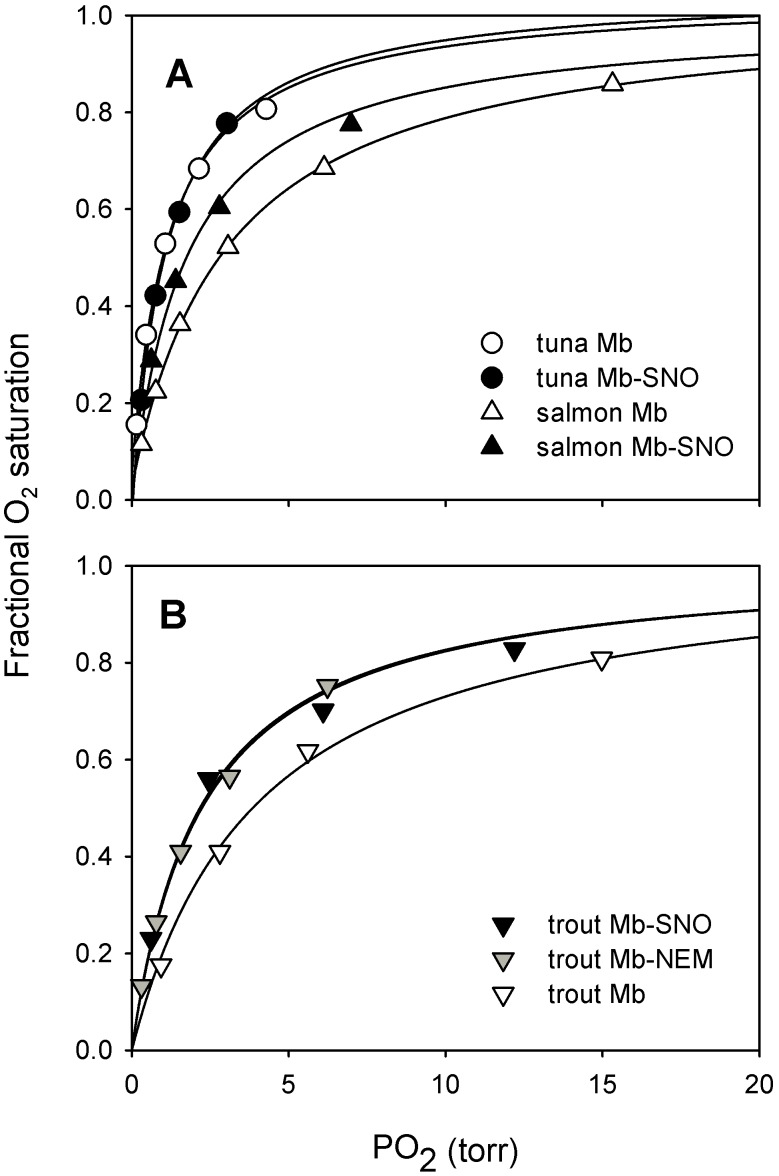
S-nitrosation increases O_2_ affinity of salmon and trout Mbs but not of tuna Mb and is functionally equivalent to modification by *N*-ethylmaleimide. A) O_2_ equilibrium curves for tuna and salmon Mb and Mb-SNO and B) O_2_ equilibrium curves for trout Mb, Mb-NEM and Mb-SNO, as indicated, measured in 50 mM Tris, 0.5 mM EDTA, pH 8.3 at 20°C. Mb-SNO data are from [9].

When salmon Mb was S-nitrosated (to ∼0.5 SNO/heme), the O_2_ equilibrium curve shifted to the left (Figure 3) and the O_2_ affinity was significantly *(P* = 0.002, n = 3) increased (*P*_50_ = 1.7±0.03 torr) compared with the unmodified protein (*P*_50_ = 2.7±0.2 torr, Table 1). When extrapolated to 1.0 SNO/heme ratio (by assuming a linear relationship between SNO/heme ratio and *P*_50_), the *P*_50_ of salmon and trout Mb-SNO was calculated to be 0.7 and 1.3 torr, respectively (Table 1) [9]. The slope of the Hill plot was close to 1 (0.96±0.05, n = 3) for the unmodified salmon Mb, indicating absence of cooperativity, but below unity for the S-nitrosated Mb (0.89±0.02, n = 3), thus confirming the functional heterogeneity of the sample containing a mixture of unmodified Mb and Mb-SNO with different O_2_ affinities. In contrast, S-nitrosation induced no change in the O_2_ affinity of tuna Mb compared with the unmodified Mb (*P* = 0.870, n = 3) (Figure 3, Table 1). Accordingly, the slope of the Hill plot was close to 1 for both unmodified (0.98±0.04, n = 3) and S-nitrosated (1.08±0.15, n = 3) tuna Mb.

**Table 1:** Measured O_2_ affinities (*P*_50_) and kinetic O_2_ dissociation rates (*k*_off_) (means ± SD; n = 3) and derived O_2_ dissociation equilibrium constants (*K*_d_) and apparent kinetic O_2_ association rates (*k*_on_) for myoglobins from yellowfin tuna, Atlantic salmon and rainbow trout in the native (Mb) and thiol-modified forms (Mb-SNO and Mb-NEM).

*Species*	*P*_50_ (torr)	*K_d_* (µM)	*k*_off_ (s^−1^)	*k*_on_ (µM^−1 ^s^−1^)
*Yellowfin tuna*				
Mb	0.9±0.01	1.6	48.4±1.3	30.3
Mb-SNO	0.9±0.1^2^	1.6	49.4±0.1^3^	-
Mb-SNO (1.0 SNO/heme)	0.9	1.6	50.4	31.5
*Atlantic salmon*				
Mb	2.7±0.2	4.8	72.9±3.0	15.2
Mb-SNO	1.7±0.03^3^ *	3.0^3^	68.6±4.4^3^	22.9^3^
Mb-SNO (1.0 SNO/heme)	0.7	1.2	64.3	53.6
*Rainbow trout*				
Mb	3.7±0.2	6.5	111.9±3.4	17.2
Mb-SNO	2.2±0.2^1^ ^,4^ *	3.9	123.1±3.9^2^	-
Mb-SNO (1.0 SNO/heme)	1.3	2.3	128.7	55.9
Mb-NEM	2.2±0.1*	3.9	144.4±1.3	37.0
Mb-NEM (1.0 NEM/heme)	1.7	3.0	155.2	51.7

Linearly extrapolated values at 1.0 SNO/heme ratio and 1.0 NEM/heme ratio are indicated. Apparent *k*_on_ values are reported for identical degrees of thiol modification. Experimental conditions: 20°C, pH 8.3.

1 0.6 SNO/heme, from [9]. ^2^0.4 SNO/heme. ^3^0.5 SNO/heme. ^4^0.6 SNO/heme. ^5^0.75 NEM/heme.

*Significantly different from the unmodified form (*P*<0.05), unpaired t-test.

Stopped flow kinetics showed little change in the O_2_ dissociation (*k*_off_) rates in the Mb-SNO derivatives compared with the unmodified Mbs from trout, salmon and tuna (Table 1). Absorbance traces were best fitted by a single exponential (Figure S1 in File S1), indicating a single transition from oxy to deoxy-Mb and that the potential cleavage of the S-NO bond by dithionite (with generation of free NO and its capture by deoxy heme) [5], [27] did not interfere with *k*_off_ measurements during the short time (<0.1 s) of the experiment. Because of the high levels of S-nitrosation of our study, any NO generated by cleavage of the S-NO bond would have resulted in evident deviations from a mono-exponential behavior. Although O_2_ association rates (*k*_on_) could not be measured directly, we could estimate apparent *k*_on_ values from measured O_2_ equilibrium dissociation constants (*P*_50_ and *K*_d_) and *k*_off_ values (Table 1). These data suggest that the increase in O_2_ affinities (i.e. the decrease in *P*_50_ values) of trout and salmon Mb due to S-nitrosation (Figure 2) is caused by ∼3-fold increases in O_2_ association (*k*_on_) rates (Table 1). Interestingly, covalent Cys modifications by NEM caused equivalent changes in *P*_50_ and *k*_on_ as in the SNO derivatives, while leaving *k*_off_ rates virtually unaffected (Table 1).

After addition of CuSO_4_, which removes NO from SNO, followed by overnight reduction with DTT, the O_2_ affinity of salmon Mb was again similar to that for the unmodified Mb (*P*_50_ = 2.6±0.2, n = 2). This finding indicates that the change in O_2_ affinity induced by S-nitrosation is reversible and due to binding and release of NO from Cys.

Free thiols in rainbow trout Mb reacted almost completely (∼0.75) with NEM, as seen from the decreased number of accessible thiols of Mb-NEM measured by 4-PDS (∼0.5 SH/heme) compared with unmodified Mb (∼1.9 SH/heme). The O_2_ affinity of rainbow trout Mb-NEM was significantly higher (*P*_50_ = 2.2±0.1 torr) than that of native rainbow trout Mb (*P*_50_ = 3.7±0.2 torr; *P*<0.001, n = 3) and was identical to that of trout Mb-SNO (∼0.60 SNO/heme) measured previously under identical conditions (2.2±0.2 torr) (Figure 3, Table 1) [9]. Trout Mb-NEM and Mb-SNO derivatives were thus functionally equivalent.

We then used trout Mb-NEM as a stable substitute for photolabile Mb-SNO in spectroscopic analyses. The UV-Vis spectra of the met, deoxy, oxy and CO forms of trout Mb-NEM complex were unchanged compared to those of the native protein (data not shown). These results suggest that complexation of Cys with NEM does not cause any significant variations in heme structure. To confirm this deduction and verify whether there may be subtle changes in heme structure induced by NEM complexation, the RR technique, sensitive to heme spin, coordination and oxidation state [28], was applied to all the forms. However, the RR spectra in the high frequency region (1300–1700 cm^−1^), showed no significant variations of Mb-NEM in the oxy, met, deoxy and CO forms (Figure S2 in File S1) compared to the unmodified protein. Therefore, the coordination and spin states of the heme are invariant upon NEM-complexation for all forms, in agreement with the UV-Vis results. Moreover, there was no variation in the frequencies of the RR iron-ligand bands, highly sensitive to the heme-ligand bond strength, as a consequence of alteration of the heme cavity structure and/or H-bonding interactions [29]. In particular, the frequencies of the ν(Fe-Im) band at 220 cm^−1^ (deoxy form), ν(Fe-C) band at 504 cm^−1^ (CO form), and ν(Fe-O_2_) band at 574 cm^−1^ (oxy form) of the Mb-NEM complex were identical to those of the native unmodified protein [24]. Since the ν(Fe-Im) frequency is sensitive to the proximal heme environment, whereas the ν(Fe-C) and ν(Fe-O_2_) frequencies are sensitive to the heme distal environment, complexation with NEM does not cause variations in heme cavity structure of the fully ligated or unligated protein.

**Figure 4:**
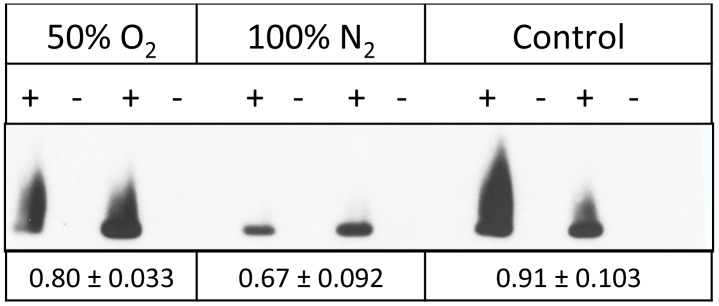
Biotin switch analysis of trout hearts indicates changes in Mb-SNO levels following incubation of trout heart preparations in oxygenated and anoxic conditions. Three freshly isolated ventricle rings were prepared from a single heart and either directly frozen (control) or incubated for 45 minutes in the presence (50% O_2_) or absence (100% N_2_) of O_2_. Following incubation, the rings were immediately frozen. Lysates were prepared from heart rings and analyzed by the biotin switch technique. Samples labeled in the presence of NEM-Biotin are marked as +, while those in which biotin was omitted (−) serve as individual controls. Image shows rings isolated from two separate hearts. Biotin-labeled Mb was quantified by densitometry and then normalized to the total protein content. The mean ± standard error Mb-SNO level (n = 5) is given below the figure.

In order to gauge the relative importance of Mb-SNO *in vivo,* we also determined the Mb-SNO content of freshly isolated trout heart preparations. Furthermore, we examined how incubating these preparations in either the absence (100% N_2_) or presence of O_2_ (50% O_2_) altered the Mb-SNO content. Figure 4 shows that Mb-SNO was found within trout hearts (control conditions). Incubation of trout hearts in a 50∶50 mix of O_2_ and N_2_ appeared to have little effect on Mb-SNO content. However, incubation in 100% N_2_ significantly (*P*<0.05) reduced Mb-SNO concentration compared to control conditions. These results indicate that Mb-SNO is found within trout heart and that its content is altered by changes in O_2_ tension.

## Discussion

A major finding of this study is that the O_2_ affinity of Atlantic salmon Mb, like that of trout Mb, increases upon S-nitrosation (i.e. *P*_50_ decreases), whereas that of yellowfin tuna does not. Therefore, allosteric changes in O_2_ affinity induced by S-nitrosation appear to be a specific characteristic of salmonid fish Mbs. The Mbs of trout and salmon are peculiar among vertebrate Mbs in that they have some of the lowest O_2_ affinity ever measured, with *P*_50_ values of 3.7 [9] and 2.7 torr at 20°C (Figure 3), respectively, whereas other fish and mammalian Mbs typically have *P*_50_∼1 torr under similar conditions [17], [30], [31]. Furthermore, besides the conserved Cys10, present in tuna Mb, the Mb of these two salmonid species contains a second Cys at position 107 (Figure 1), located at the interface between the G and H helices of the Mb, far from the heme pocket. These findings indicate that the tertiary allosteric effect of S-nitrosation found in salmonid Mbs may to be associated with a low O_2_ affinity and with the presence of Cys107. Modifications at this particular Cys107 residue may then be able to induce changes in the tertiary structure of the Mb that affect heme O_2_ affinity. These changes must be different from those seen in crystals of tuna Mb-SNO, where S-nitrosation takes place at Cys10 [18]. In support of this conclusion, S-nitrosation has no effect on the O_2_ affinity of either tuna Mb (Figure 2) or carp Mb1 and Mb2 [14], where Cys107 is absent (Figure 4), and despite the presence of other reactive Cys residues potentially available for SNO formation (Figure 1). Thus, the notion that the physiological effects of S-nitrosation on protein functions are exerted through selective rather than general thiol modifications applies for the Mb as well as for the other proteins in which these effects have been studied [1].

Interestingly, the extrapolated *P*_50_ of the S-nitrosated derivative at 1.0 SNO/heme of salmon and trout Mbs (∼0.7 torr and ∼1.3, respectively) are remarkably close to the *P*_50_∼1 torr typical of other fish Mbs [14], [17], [30], including tuna (Table 1; Figure 3), whereas in the unmodified form these two Mbs have higher *P*_50_ values (i.e. low affinity). In trout Mb, covalent attachment of NEM to Cys residues caused a similar increase in the O_2_ affinity as S-nitrosation (Table 1; Figure 2), suggesting that similar structural changes are induced by both these thiol modifications. Although the crystallographic structure of trout Mb has not been solved yet, our data indicate that its unusually low O_2_ affinity may derive from some structural constraints that are relieved when the protein become S-nitrosated at Cys107. Spectroscopic data from this study showed no changes in either the UV-Vis or RR spectra of the Mb-NEM complex (functionally identical to Mb-SNO) compared with the native protein. In particular, there was no variation in the frequencies of the RR Fe-ligand bands which are highly sensitive to heme cavity structure and H-bonding interactions, hence complexation of Cys with NEM and most likely S-nitrosation did not cause any significant perturbation of the proximal or distal sides of the heme cavity. These findings indicate that the effect of S-nitrosation on the O_2_ affinity of trout Mb is not to increase the strength of the H-bond between the heme-bound O_2_ and the distal His in the fully ligated protein [24].

That the O_2_ equilibrium curve of Mb-SNO is left-shifted compared to that of the unmodified Mb indicates that S-nitrosation decreases the O_2_ tension required to achieve a given saturation level but without affecting the heme-ligand stability of the fully oxygenated protein. Our kinetic data suggest that the left-shift of the O_2_ equilibrium curves induced by S-nitrosation in trout and salmon Mb is mainly due to an increase in the rate of O_2_ association (Table 1). For the oxygenation reaction, the dissociation equilibrium constant, *K*, equals the ratio between the O_2_ dissociation (*k*_off_) and association rates (*k*_on_) [31], and therefore any increase in O_2_ affinity is caused by a decrease in *k*_off_ and/or an increase in *k*_on_. O_2_ dissociation *k*_off_ rates measured by stopped flow did not change noticeably upon S-nitrosation or S-derivatization with NEM, whereas derived apparent association *k*_on_ rates increased approximately 3-fold with S-nitrosation (Table 1) to values similar to those found for the Mbs from other species [17]. As a consequence, tertiary structural changes caused by S-nitrosation at Cys107 in trout and salmon Mbs appear to ease the access of O_2_ to the heme cavity compared to the unmodified protein and to render these Mbs functionally more similar to other Mbs. High *k*_off_ (range ∼73–112 s^−1^) and low *k*_on_ (range ∼15–17 µM^−1 ^s^−1^) values account for the low O_2_ affinities of native trout and salmon Mbs (Table 1) compared with high-affinity Mbs from other fish species (*k*_off_ range ∼34–62 s^−1^, *k*_on_ range ∼30–44 µM^−1 ^s^−1^) [17]. Structural analyses of the trout or salmon Mb protein in the unmodified and S-nitrosated forms will be needed to solve the molecular mechanisms underlying these effects.

The tertiary SNO-dependent allosteric effect described in this study shows that a specific covalent modification at Cys107, such as S-nitrosation, is necessary to alter heme reactivity of monomeric Mb. Because of the very low (n-molar) levels of Mb in the S-nitrosated form *in vivo*, this allosteric mechanism of Mb function would regulate O_2_-dependent delivery of physiologically relevant amounts of NO but would not be able to modulate NO-dependent O_2_ delivery to any appreciable extent. In addition, the SNO-dependent covalent tertiary effect described here contrasts with a postulated lactate effect on Mb [32] that has been recently re-proposed [33], but that has not been confirmed by us [10] or other investigators.

The finding that S-nitrosation appears to be favored by oxygenation is supported by our observations of Mb-SNO content within trout hearts. Using the biotin switch technique, we were able to demonstrate that Mb-SNO was found within untreated trout hearts. Importantly, it was observed that incubating heart rings in anoxic conditions reduced the Mb-SNO content. These results demonstrate that Mb-SNO is found *in vivo* and thus these mechanisms may be of significance *in vivo*. But perhaps, more significantly one can see that SNO content of Mb is linked to O_2_ tension. These experiments, however, do not give insight into the kinetics of S-nitrosation and denitrosation *in vivo* as hearts were incubated for 45 minutes in 100% N_2_. Therefore, we cannot make direct conclusions as to whether this is a dynamic regulation that may be important in the rapid matching of O_2_ delivery and demand. Potentially, however, this mechanism of hypoxia-induced NO delivery may contribute to increase myocardial efficiency [11] in salmonid fish during intense swimming (i.e. during spawning migrations), where the heart is likely to become severely hypoxic. Whether and why only salmonid fish would benefit from this additional way of regulating NO homeostasis in the heart remains to be investigated.

## Supporting Information

File S1 **Combined file of supporting figures. Figure S1.** Kinetic traces of the reaction between rainbow trout Mb-SNO (0.4 SNO/heme) and 20 mM dithionite (after mixing) measured by stopped flow show mono exponential behavior. Traces were measured at 430 nm (A), 418 nm (B) and 420 nm (C) at 20°C, in deoxygenated 100 mM Tris, 0.5 mM EDTA pH 8.3. Mono exponential fitting of the traces is indicated. **Figure S2.** Trout Mb (left panel) and its complex with NEM (right panel) have identical high-frequency RR spectra. Mb: Met in 0.1 M MES at pH 6.0, oxy in 0.1 M Tris-HCl at pH 7.6, 0.5 mg/mL DDT, CO complex in 0.1 M Tris-HCl at pH 7.6, deoxy in 0.1 M Tris-HCl at pH 7.6; Mb-NEM: all forms in 0.05 M Hepes at pH 7.2. Experimental conditions: (met, oxy and CO) 413.1 nm excitation wavelength, 1 cm^−1^ spectral resolution, 5 mW laser power at the sample (met, oxy), 1 mW laser power at the sample (CO); (deoxy) 441.6 excitation wavelength, 1 cm^−1^ spectral resolution, 15 mW laser power at the sample. The intensities are normalized to that of the ν_4_ band (not shown). The assignment of the RR bands is given in ref. [24].
(DOCX)
