# Supplementary figures and images for: Oxygen-Linked S-Nitrosation in Fish Myoglobins: A Cysteine-Specific Tertiary Allosteric Effect
Source: PLoS One. 2014 May 30;9(5):e97012. doi: 10.1371/journal.pone.0097012 (PMC4039430; doi:10.1371/journal.pone.0097012)

**Figure S1**

**Figure S2**


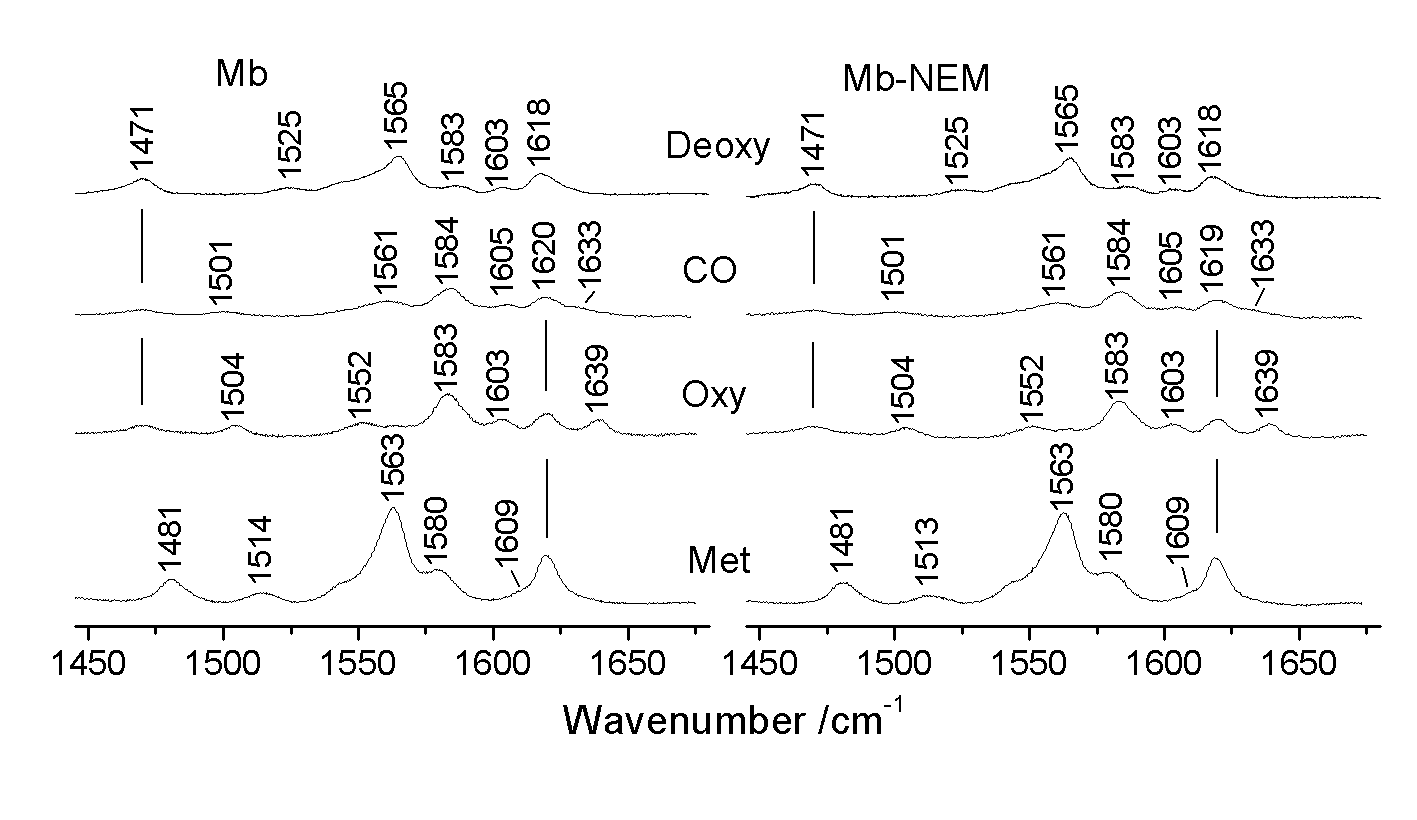

Supplement: File S1 — Combined file of supporting figures. Figure S1. Kinetic traces of the reaction between rainbow trout Mb-SNO (0.4 SNO/heme) and 20 mM dithionite (after mixing) measured by stopped flow show mono exponential behavior. Traces were measured at 430 nm (A), 418 nm (B) and 420 nm (C) at 20°C, in deoxygenated 100 mM Tris, 0.5 mM EDTA pH 8.3. Mono exponential fitting of the traces is indicated. Figure S2. Trout Mb (left panel) and its complex with NEM (right panel) have identical high-frequency RR spectra. Mb: Met in 0.1 M MES at pH 6.0, oxy in 0.1 M Tris-HCl at pH 7.6, 0.5 mg/mL DDT, CO complex in 0.1 M Tris-HCl at pH 7.6, deoxy in 0.1 M Tris-HCl at pH 7.6; Mb-NEM: all forms in 0.05 M Hepes at pH 7.2. Experimental conditions: (met, oxy and CO) 413.1 nm excitation wavelength, 1 cm−1 spectral resolution, 5 mW laser power at the sample (met, oxy), 1 mW laser power at the sample (CO); (deoxy) 441.6 excitation wavelength, 1 cm−1 spectral resolution, 15 mW laser power at the sample. The intensities are normalized to that of the ν4 band (not shown). The assignment of the RR bands is given in ref. [24]. (DOCX) [file pone.0097012.s001.docx]
